# Case Report: Four cases for cariprazine and alcohol use disorder

**DOI:** 10.3389/fpsyt.2026.1772314

**Published:** 2026-02-25

**Authors:** Sreten Vicentic

**Affiliations:** 1 Clinic of Psychiatry, University Clinical Center of Serbia, Belgrade, Serbia; 2 Faculty of Medicine, University of Belgrade, Belgrade, Serbia

**Keywords:** alcohol dependence, cariprazine, case report, cognitive symptoms, D3 receptors

## Abstract

**Background:**

Alcohol can diminish individual health from the fetus to old age and affects a wide range of structures and processes in the central nervous system. The role of dopamine D2 and D3 receptors in addictive diseases is the subject of many years of research. Among many psychotropic medications, cariprazine actually preferentially binds to D3 receptors, and its binding is stronger than that of any other antipsychotic, and even that of dopamine itself. Some data indicate that cariprazine and its effects associated with D3 partial agonism, could improve the domain of cognitive and depressive symptoms, as well as in the sphere of motivation, reward and cravings reduction.

**Cases presentations:**

This case report describes four individual cases of patients who were alcohol addicts, with multiple hospitalizations during their treatment of dependence. All four patients were treated during the years with many different pharmacologic protocols, involving antipsychotics (olanzapine, quetiapine), mood stabilizers (carbamazepine, lamotrigine), anxiolytics (clonazepam, bromazepam), SSRI antidepressants (fluoxetine, paroxetine, escitalopram), but without achieving of the alcohol abstinence. Only by adding of cariprazine in the protocol, with almost total exclusion of other phychopharmacs, they achieved stable alcohol abstinence. Similarly, across all four cases, a marked improvement in depressive and cognitive symptoms was observed, and although each patient had been treated with antidepressants for many years, meaningful improvement occurred only after the introduction of cariprazine.

**Conclusion:**

The consistent achievement of full abstinence across all four patients suggests that it may have potential relevance in the management of alcohol use disorder (AUD). However, in the future, well conducted and highly controlled studies are needed to explore a potential cariprazine’s role and its place in the management of alcohol dependence.

## Introduction

It is well known that alcohol can diminish individual health from the fetus to old age. Alcohol affects a wide range of structures and processes in the central nervous system. Associated with personality characteristics, is causal factor for injuries, absenteeism, many somatic diseases, as well some types of cancers (1). Alcohol contributes in about 100 million disability-adjusted life-years across the world, while some data show there were more than 60 million cases of alcohol use disorder (AUD) globally (2). The fact that the treatment process is very difficult and complex, and that there are still no sufficiently effective psychopharmaceuticals that would guarantee the reduction of cravings and the establishment of permanent abstinence, is of additional concern. Cravings refers to the subjective need and desire to use substances. Further, the individual experience of cravings is perceived to be the first aspect of and driving force behind substance use. Unfortunately, there are still no specific guidelines for the effective pharmacological treatment of cravings (3).

The role of dopamine D2 and D3 receptors in addictive diseases is the subject of many years of research (4). D2 receptors are linked with mesocortical and mesohippocampal dopamine systems, the D3 receptors are connected with the ventral forebrain mesolimbic system (5). That position of the D3 receptors is very suitable to influence reward, emotion, motivation and drug seeking and relapse mechanisms. Psychoactive substances increase dopamine in the nucleus accumbens, a major component of the reward system, due to expression of D3 receptors in this specific localization, there is huge interest about the potential role of D3 receptors in drug reward and addictive processes. Antagonism or partial agonism of DRD3 has been shown to reduce the alcohol deprivation effect (6). Some results indicate that a year of voluntary alcohol drinking and in high alcohol drinking was connected with higher D3 gene expression in the striatum (7).

Among many psychotropic medications, cariprazine actually preferentially binds to D3 receptors, and its binding is stronger than that of any other antipsychotic, and even that of dopamine itself (8). Considering the very high affinity of dopamine for D3 receptors, the otherwise low affinities of antipsychotics, with the exception of cariprazine, render them incapable of blocking D3 receptors in the presence of dopamine in the living brain (9).

## Cases presentation

### Case 1

Mr. A is a 46-year-old married man with two children. He completed high school and works as a caterer, currently employed as a waiter in a restaurant. His psychiatric history spans approximately eight years and includes outpatient treatment as well as multiple psychiatric hospitalizations for alcohol dependence and comorbid depression. His family history is negative for psychiatric or addiction-related disorders.

He first began consuming alcohol in high school, at the age of 18, mainly beer, 1-1.5 L a few times weekly, initially in social settings. By the end of high school and especially after starting work at age 18, his drinking intensified, he drunk 4–7 L of beer daily. Over time he developed clear signs of alcohol dependence, like noticeable decrease in tolerance and control over his drinking, as well attempts to quit on his own were followed by pronounced withdrawal symptoms, including sweating, tremors, leg pain, tension, nervousness. The longest periods of abstinence were about one month. Regarding to relapses, no psychosocial changes or stressors occurred during treatment, moreover his family situation was very stable and supporting.

In addition to alcohol addiction, his main psychiatric symptoms included anxiety, insomnia, and listlessness. Over the years he was treated with various psychiatric regimens, typically combinations of mood stabilizers (carbamazepine, up to 600 mg/day), antidepressants (fluoxetine 20 mg/day), and antipsychotics. Antipsychotics—initially olanzapine for several years (5 mg/day), later quetiapine (up to 100mg/day) — were used with the aim of reducing cravings, but neither contributed to achieving abstinence.

During his most recent hospitalization in September 2023, cariprazine was introduced, starting at 1.5 mg/day and later increased to 3 mg/day. Since the initiation of cariprazine, he has remained abstinent for two years. His current treatment includes cariprazine (3 mg), valproate (1500 mg/day), and a low dose of an SSRI antidepressant, fluoxetine, 10 mg/day. He regularly attends outpatient follow-ups every two months, is steadily employed, and is well integrated into both his family life and work environment.

### Case 2

Mr. B is an employed engineer in a state-owned company. He is divorced, lives with his mother, and has two adult children with whom he maintains a good relationship. His history of alcohol dependence spans approximately six years, during which he was hospitalized three times, most recently in May 2023. Thereafter, he did not attend outpatient follow-ups regularly. His family history is notable for alcoholism in his grandfather, who was never treated, this fact could represent moderate risk factor.

His problematic drinking began in 2017, initially through heavy consumption of hard liquor, about 500ml of spirits, usually in social settings with friends. Over time, his drinking escalated, and he began drinking alone at home, daily, mostly used was vodka. He reports that even a single glass of 50 ml of vodka was enough to rapidly lose control and consume up to 500 ml of spirits, with 40% of an alcohol strength. Episodes of amnesia and alcohol-related aggression are consequences.

In previous years, Mr. B was treated with quetiapine (200 mg/day), carbamazepine (up to 600 mg/day), various antidepressants (fluoxetine 20 mg/day or paroxetine 20 mg/day), anxiolytics (clonazepam 2 mg/day), and hypnotics for insomnia (zolpidem 10 mg/day). During his most recent hospitalization in 2023, his treatment was optimized, antidepressant, anxiolytic and hypnotic were excluded, and his current medication regimen includes cariprazine 1.5 mg/day and carbamazepine 400 mg/day. Since that time, he has remained completely abstinent. He is active, socially engaged, and continues to maintain healthy relationships with his children.

### Case 3

Mr. C is a 56-year-old retired police officer who lives with his wife and has two children—a son and a daughter. The beginning of alcohol consumption was in age of 20, few times per week, about 50 ml of spirits, but he sought psychiatric help four years ago due to long-standing alcohol misuse, in social setting or alone, which had intensified after his participation in combat zones, in his age of 35. Over time, his alcohol tolerance declined and he developed significant lethargy and severe insomnia. His daily alcohol intake reached approximately 500 ml of spirits, about 40% of an alcohol strength. Family history of consumption is negative.

In addition to alcohol dependence, he presented with multiple psychiatric symptoms: strong cravings, mood instability, suicidal thoughts, tension, irritability, and increasingly interpretive thinking that bordered on relationship- and persecutory- delusions. He also experienced occasional auditory hallucinations. He underwent three psychiatric hospitalizations and experienced multiple relapses.

Following his first hospitalization, he was treated with antidepressants (paroxetine 20 mg/day), anxiolytics (lorazepam 7.5mg/day), a hypnotic (zolpidem 10 mg/day), and olanzapine 10 mg/day. After the second hospitalization, quetiapine 200 mg/day was added. However, these regimens did not alleviate his symptoms or help him achieve abstinence.

During his most recent hospitalization in March 2023, his treatment was modified, by adding cariprazine, with the starting dose of 1.5 mg/day. Since then, he has remained completely abstinent. His current medication includes cariprazine 3 mg/day, trazodone 50 mg at night, and an anxiolytic as needed (bromazepam 1.5 mg). His delusional symptoms and auditory hallucinations have fully resolved, and he is now functioning well in daily life.

### Case 4

Mr. D is a 35-year-old high school graduate who works as a motor vehicle driver. He is single and lives with his father. He began drinking alcohol at age 20, firstly drank beer, about 1–2 L during weekend, few years later he started to drink spirits, about 100 ml four times per week in average, than in age of 25 he drank daily, about 200–300 ml of spirit, in social setting with his friends or alone. He struggled with dependence for many years, presented with decrease in tolerance toward alcohol and loss of control over his drinking, as well withdrawal symptoms, hands shaking, sweating, leg pain, insomnia. His first psychiatric hospitalization occurred in November 2015, followed by a second in December 2018, each at different institutions, due to diagnosis of alcohol dependence. His main complaints included strong alcohol cravings, anxiety, various fears, loss of control during drinking episodes, guilt, and significant sleep disturbances (initial, intermittent, and terminal insomnia). Hospitalizations resulted in temporary periods of abstinence—nine months after the first and one month after the second. He has been treated at the current institution since 2020 and has required five additional hospitalizations due to repeated inability to maintain abstinence. Lately, he also reported sadness related to his mother’s illness and his own perceived failures in trying to overcome alcohol dependence. Over the past five years, he changed more than ten jobs, consistently losing employment because of alcohol-related issues. His family history is notable for his father, who is a treated alcoholic, and this fact represents higher risk factor for alcohol consumption.

Prior treatments included anxiolytics (bromazpam 9 mg/day), olanzapine 5 mg/day, mood stabilizers (carbamazepine 800 mg/day), and later a combination of quetiapine 150 mg/day, antidepressants (escitalopram 10 mg/day) and lamotrigine (100–200 mg/day). Despite these efforts, he repeatedly relapsed within 1–2 months after each treatment attempt.

During his most recent hospitalization in February 2024, his treatment regimen was modified, by adding cariprazine and with exclusion of quetiapine, lamotrigine, escitalopram and bromazepam. Since then, he has maintained continuous abstinence for 20 months. He has secured stable employment, attends outpatient check-ups regularly, and reports no relapse episodes. His current medication includes cariprazine 3 mg/day and valproate 1000 mg/day.

## Discussion

In the cases presented above, cariprazine was used outside of its approved and clinically studied indications. Nevertheless, the consistent achievement of full abstinence across all four patients suggests that it may have potential relevance in the management of alcohol use disorder (AUD).

There were no possible side effects of cariprazine treatment in any of the cases.

The first case involved a patient with a lifelong history of alcoholism who had failed multiple prior pharmacological treatments. Only after initiating cariprazine did he achieve stable abstinence, without experiencing adverse effects. A similar pattern appears in Case 4: despite many years of alcohol dependence and unsuccessful attempts with olanzapine and quetiapine, abstinence was reached only after cariprazine was introduced. The two other cases involve younger patients with shorter disease duration, where cariprazine was equally successful in treating AUD. Notably, the patient in Case 2 remained on 1.5 mg/day; all others showed greater improvements in abstinence after increasing the dose from 1.5 mg to 3 mg/day.

The potential role of cariprazine in reducing alcohol or substance use may relate to its very high affinity for D3 receptors (10, 11) which are abundant in the brain’s reward circuitry and are deeply involved in addiction pathophysiology (11–13). This could imply that cariprazine can show the effects usually associated with D3 partial agonism, which are improvements in the domain of negative, cognitive and depressive symptoms, as well as in the sphere of motivation and reward (14). Beyond the cases reported here, supporting evidence comes from preclinical studies: in animal models, cariprazine reduced the rewarding effects of cocaine and prevented relapse, and the abuse preventing potency of cariprazine was ~20-fold higher than that of aripiprazole (15). Preliminary clinical observations also suggest that cariprazine may be useful for patients with comorbid substance use disorders. A real-world evidence study and various case reports are available to underscore cariprazine’s efficacy on addiction symptoms (16–21). In a study by Szerman et al, authors examined the use of cariprazine for treating dual disorders, specifically comorbid substance use disorder (SUD) and schizophrenia (20). Cariprazine treatment led to significant improvements in schizophrenia symptoms, along with a decrease of cannabis use (20). These findings are further supported by case reports describing cariprazine’s efficacy in reducing the craving and substance use in patients consuming methamphetamine, cocaine, cannabis, alcohol and tobacco (19–24). In consequence, current guidelines suggest cariprazine and other partial agonists as first line treatment in maintenance settings and as second line in acute settings of substance use disorder comorbidities (12, 22, 23). In a functional MRI study examining brain responses to brief cocaine-related cues (500 ms), a group of cocaine-dependent patients treated with cariprazine 3 mg/day (n = 4) was compared with an unmedicated, demographically similar control group (n = 14). The findings showed that cariprazine markedly reduced cue-induced limbic activation in the treated patients (24).

Across all four cases, a marked improvement in depressive symptoms was observed. Although each patient had been treated with antidepressants for many years, meaningful improvement occurred only after the introduction of cariprazine. It remains uncertain to what extent this improvement reflects the direct pharmacological properties of cariprazine, the effects of sustained alcohol abstinence, or an interaction of both factors. Nevertheless, the antidepressant effects of cariprazine are well documented in the literature, and the medication is approved for bipolar depression as well as for adjunctive treatment of major depressive disorder in the United States and other countries (25). Its efficacy has been demonstrated in four bipolar depression studies and in several MDD add-on studies, all of which confirmed significant improvements in depressive symptoms (26). The antidepressant properties of cariprazine are thought to arise primarily from its partial agonism at dopamine D3 and D2 receptors, serotonin 5-HT1A receptors, and its antagonistic activity at 5-HT2B and 5-HT2A receptors (27).

The Cases 1 and 2, belong to the so called category of AUD without comorbid psychosis, so the clinical rationale for the prescribing of antipsychotics in the context of AUD lies in their above mentioned effects at dopamine D3 and D2 receptors. Many studies suggest that atypical antipsychotic agents may be beneficial for the treatment of alcohol dependence, as olanzapine (28, 29) and quetiapine (30, 31).

In Case 3, psychotic features—including delusions, occasional auditory hallucinations, and suicidal ideation—resolved completely within one month of starting cariprazine, alongside full abstinence. It is not surprising that cariprazine alleviated positive symptoms such as delusions and hallucinations, given its well-established efficacy in schizophrenia. Cariprazine’s antipsychotic effects have been demonstrated across six clinical studies, and subgroup analyses specifically examining positive symptom domains consistently show significant improvements (26). Real world effectiveness studies further support these findings (16, 32–35).

In the presented cases patients also improved related to their cognitive functioning, according to improvements in the area of remembering, speaking and overall communication, as well understanding and especially better concentrating. Consecutively, better cognitive functioning resulted in less feel of exhaustion, less stressed and reduction of depressive symptoms. The dopamine D3 receptor has been identified as a key target for cognitive enhancement, and cariprazine’s partial agonism at D3 is thought to contribute to potential cognitive benefits. This effect has been supported by subgroup analyses from several clinical studies, indicating that cariprazine may indeed improve cognitive performance in relevant patient populations (29). Further, beside the many advantages of randomized controlled trials (RCTs), one of the biggest concerns is generalizability, as populations involved in these trials can significantly diverge from those seen in actual clinical practices (36). Similarly, patients with AUD are excluded of clinical trials, and the therapeutic product does not seem feasible in real-world settings for these population (37, 38). Therefore, in psychiatry, especially in the field of addiction, information from evidence-based medicine, such as case reports, are highly valuable. The most recent and relevant studies of Schröder et colleagues (39–41), were focused in the possibility of alcohol–medication interactions, and shown that a significant number of patients with AUD receive medications with the potential to interact with alcohol. The use of interaction checks to assess the appropriateness of medications for the addiction setting could be beneficial in improving medications safety for the patients with AUD.

## Conclusion

Our cases show that cariprazine could represent a potential successful strategy in patients with severe AUD and other psychiatric diagnoses. Some psychiatric symptoms in these patients were partially resolved with other medications, but only cariprazine decreased cravings for alcohol. This fact could contribute potentially to the rationale for cautiously using cariprazine off‐label for AUD in patients without comorbidities, who have not responded to other medications such as naltrexone, quetiapine, olanzapine. Additional fact, that cariprazine is associated with fewer metabolic side effects compared to other antipsychotics, could positively influence the compliance (42). However, in the future, well conducted and highly controlled studies are needed to explore a potential cariprazine’s role and its place in the management of alcohol dependence.

## Data Availability

The raw data supporting the conclusions of this article will be made available by the authors, without undue reservation.
